# From Cellulosic Based Liquid Crystalline Sheared Solutions to 1D and 2D Soft Materials

**DOI:** 10.3390/ma7064601

**Published:** 2014-06-18

**Authors:** Maria Helena Godinho, Pedro Lúcio Almeida, João Luis Figueirinhas

**Affiliations:** 1 CENIMAT/I3N, Departamento de Ciência dos Materiais, Faculdade de Ciências e Tecnologia, FCT, Universidade Nova de Lisboa, 2829-516 Caparica, Portugal; 2 Área Departamental de Física, Instituto Superior de Engenharia de Lisboa, Instituto Politécnico de Lisboa, R. Conselheiro Emídio Navarro, 1, 1950-062 Lisboa, Portugal; E-Mail: palmeida@adf.isel.pt; 3 Departamento de Física, Instituto Superior Técnico, Universidade de Lisboa, Av. Rovisco Pais, 1049-001 Lisboa, Portugal; 4 Centro de Física da Matéria Condensada da Universidade de Lisboa, Av. Prof. Gama Pinto 2, 1649-003 Lisboa, Portugal; E-Mail: figuei@cii.fc.ul.pt

**Keywords:** cellulose-based liquid crystals, films, fibers, Nuclear Magnetic Resonance (NMR), electro-optical characteristics

## Abstract

Liquid crystalline cellulosic-based solutions described by distinctive properties are at the origin of different kinds of multifunctional materials with unique characteristics. These solutions can form chiral nematic phases at rest, with tuneable photonic behavior, and exhibit a complex behavior associated with the onset of a network of director field defects under shear. Techniques, such as Nuclear Magnetic Resonance (NMR), Rheology coupled with NMR (Rheo-NMR), rheology, optical methods, Magnetic Resonance Imaging (MRI), Wide Angle X-rays Scattering (WAXS), were extensively used to enlighten the liquid crystalline characteristics of these cellulosic solutions. Cellulosic films produced by shear casting and fibers by electrospinning, from these liquid crystalline solutions, have regained wider attention due to recognition of their innovative properties associated to their biocompatibility. Electrospun membranes composed by helical and spiral shape fibers allow the achievement of large surface areas, leading to the improvement of the performance of this kind of systems. The moisture response, light modulated, wettability and the capability of orienting protein and cellulose crystals, opened a wide range of new applications to the shear casted films. Characterization by NMR, X-rays, tensile tests, AFM, and optical methods allowed detailed characterization of those soft cellulosic materials. In this work, special attention will be given to recent developments, including, among others, a moisture driven cellulosic motor and electro-optical devices.

## 1. Introduction

**Figure 1:**
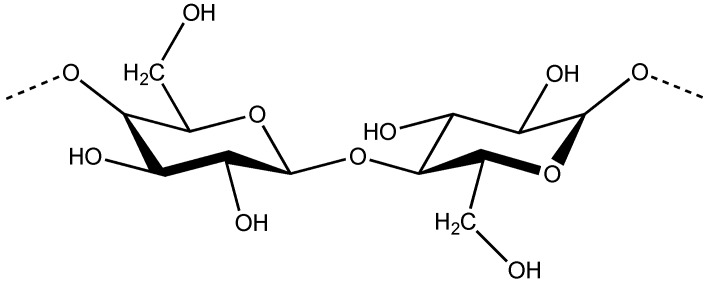
Chemical structure of the repeating unit of cellulose (dimer cellobiose).

Cellulose, the main constituent of plant cell walls, is a linear polysaccharide (Figure 1), which is mainly used to produce paper. However, in this work we are more interested in soft materials obtained from converted cellulose, not only into nano rods, but also in cellulose derivatives. In 1976, Gray reported that concentrated solutions of cellulose derivatives could self-assemble into ordered phases [1]. Those liquid crystalline phases displayed colors that changed with concentration and viewing angle, which were attributed to a cholesteric structure. The maximum peak wavelength (λ_0_) reflected by the samples for incident light normal to the surface may be expressed as, λ_0_ = *n*_e_*P*cosθ, where *n*_e_ is the average refractive index; *P* the helical pitch; and θ the angle between the light propagation direction and the helix axis [2]. The values of λ_0_ may, therefore, be tuned by altering the helical pitch or the average refractive index of the chiral nematic material. Some theories were proposed to interpret the variation of the pitch with polymer concentration. For example, the theory proposed by Kimura *et al*. [3] predicts a linear dependence of the reciprocal pitch against ϕ(1 – ϕ/3)/(1 – ϕ)^2^, where ϕ is the polymer concentration, which is in fairly good agreement with the experiments [4]. Due to cellulose relatively stiff cellulose backbone, which forces a parallel orientation of the chains, a wide range of cellulose derivatives was found to form both lyotropic and thermotropic chiral nematic liquid crystals [5]. The selective light reflection, as well as the optical turbidity of the liquid crystalline cellulose derivatives solutions can be tuned, not only by the application of external fields [6,7], but also by the addition of aqueous salt solvents [8] and by playing with solvent/cellulose derivative systems [9,10,11]. The dynamic control of the cholesteric coloration and optical clarity of lyotropic aqueous cellulose derivatives was more recently achieved by using various N-alkyl-substituted methylimidazolium salts and a weak electric field [12,13]. The electro-optical behavior of the system was interpreted in terms of the salt-containing liquid-crystalline system and the medium viscous electrolytic and high resistance characteristics [12]. In order to improve the optical characteristics of the solutions the treatment of surfaces, as well as the use of solvents that decrease the viscosity of the liquid crystalline cellulose solutions was investigated [14]. Many efforts were done to trap the chiral nematic structure into iridescent solid films. Principal attempts refer the use of solvents that can undergo polymerization and also the use of UV sensitive substituents [15,16,17,18]. Lasing in solid cross-linked cholesteric cellulose based films with high reflectivity doped with fluorescent dyes was also recently evidenced in literature [19]. The fabrication of cellulosic spheres by microfluidics, with iridescence was also described in literature [20]. In addition to cellulose derivatives liquid crystalline phases it was also found that Nano crystalline cellulose (NCC) films were capable of reflecting colored light if prepared from liquid-crystalline cellulose suspensions. NCC liquid crystal templates were used to generate new photonic materials, which can combine mesoporosity with long-range chiral ordering [21,22]. Moreover the incorporation of NCC rods into composite materials results into systems with enhanced mechanical properties, which have been investigated systematically in literature [23]. NCC rods seemed the perfect material to incorporate in cellulosic water-soluble matrices because of their intrinsic hydrophilic character [24]. Many studies were performed and described in literature that concerns the preparation of films from NCC/polymer mixtures in aqueous media [25], more recently nano cellulose paper was applied as substrate and gate dielectric to produce flexible field effect transistors [26] and also electro-optical sensors [27]. 

**Figure 2:**
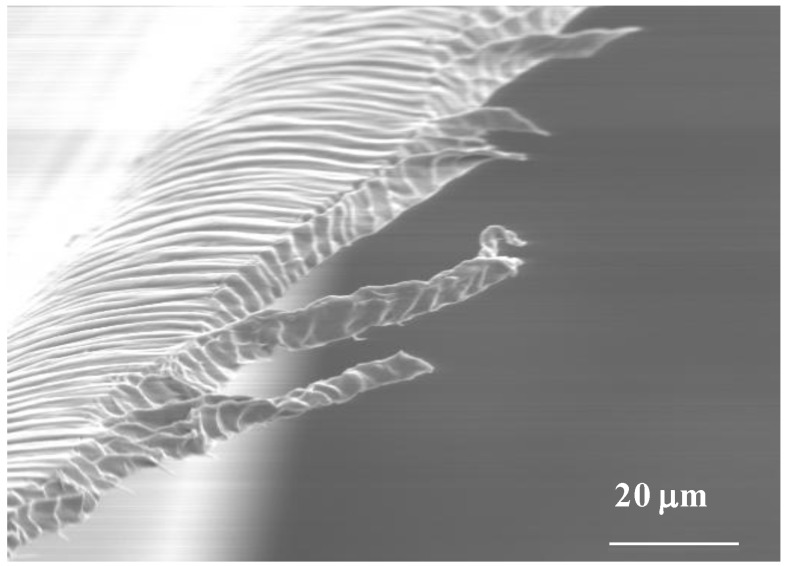
Scanning electron microscopy micrograph of a free-standing cellulosic film prepared from a sheared liquid crystalline solution.

In addition to that, sheared cellulose derivatives casted solid films, with a complex network of defects, which are easily identified by polarizing optical microscopy (POM), under cross polars, were also obtained and studied [28]. Various reports were published in order to correlate the defects appearance and the rheological behavior of the precursor solutions but some fundamental questions are not yet answered (Figure 2). X-rays results showed that the cholesteric order can be destroyed in sheared films and that a bundle of warped helicoidal fiber-like structures where the cellobiose block spins around the axis of the fiber can develop. The bundles should have fibers with both the levogyre and dextrogyre arrangements, with equal probabilities, and have a residual orientation along the shear direction [29]. 

Atomic force microscopy (AFM) measurements revealed that sheared films topographical features could be affined by playing with the films processing conditions [28]. It was demonstrated that the films prepared from liquid crystalline solutions can show two periodic structures, primary and secondary set bands. The former consisting of bands perpendicular to the shear direction while the latter has the bands slightly tilted from that direction. An out-of-plane angle variation of the sinusoidal molecular orientation was also reported [29,30]. The anisotropic nature of cellulosic solutions give them also unique characteristics that has impact, for example, in the way the viscosity changes with polymer concentration. For low polymer concentrations, while in the isotropic phase, the viscosity of the solutions increases with the increase of polymer fraction. However, for higher polymer concentrations, corresponding to the formation of the liquid crystalline phase, the increase in concentration results in a decrease of the viscosity. This was attributed to the spontaneous orientation of the mesogenic cellulose segments above the critical concentration [31]. The viscosity drop, associated to the liquid crystalline phase appearance, is an important effect that one can take profit from when working with relatively high concentrated cellulose based solutions. The characteristics of the solutions make them suitable for preparing shear-casting films with unique characteristics that allowed the preparation of alignment layers to liquid crystals, as well as refractive gratings [24,27].

**Figure 3:**
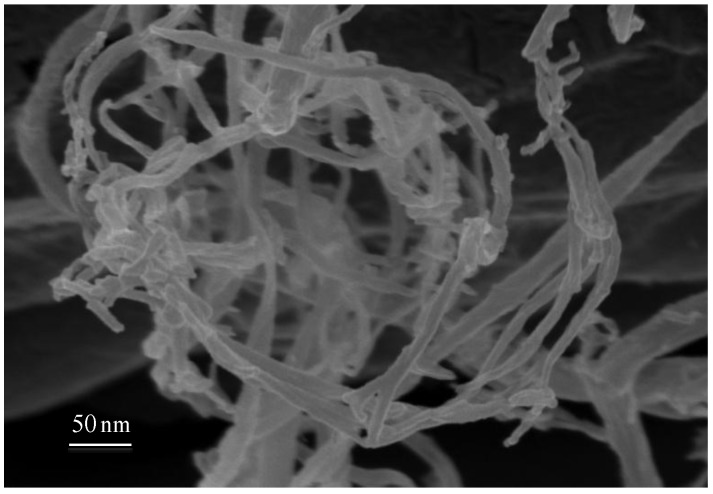
Scanning electron microscopy micrographs of a nanocellulose fibers network mat obtained from cotton after acid hydrolysis.

Electrospinning as well as cellulose acid hydrolysis (Figure 3) can be used to produce fibers from cellulose based liquid crystalline solutions, with diameters ranging from few nanometers to hundreds of microns, which can be at the origin of mats with very high surface areas [32]. The most interesting result obtained from cellulosic liquid crystals by using the electrospinning technique, was the generation of jets and fibers with intrinsic curvature, which arise from stiff line defects (disclinations) that develop during processing. Due to the intrinsic curvature of the system micro-jets swinging as well as micro/nano fibers winding and overwinding, self-motions could be observed and controlled by adjusting temperature and electrospun experimental parameters. These cellulose-based materials not only mimic at the micro/nano scale the helical shapes observed, for example, in tendrils but also their motion and mechanical adaptability could be reproduced. The same mechanism was in action for all these systems providing that the same physical model, proposed for elastic filaments with intrinsic curvature, could be applicable. The cellulose-based non-woven mats produced were expected to play a significant role in the generation of nano- and microdevices, because of their capacities as separation media, actuators, and host materials for drug delivery [31,32,33]. 

In this work, we aim to review snapshots of research carried out on cellulosic-based liquid crystalline systems, which give insights into the relation between structure/properties on sheared casted films and fibers and networks of cellulose nanorods (not organized in an iridescent cholesteric structure)/Liquid crystal systems. In particular we will focus on the use of several techniques, which include solid state NMR, Rheo-NMR, MRI, X-rays, AFM, and mechanical essays. To illustrate the potential applications of the fibers and films produced from liquid crystalline cellulose networks we refer their use to build a soft sensitive moisture motor and electro-optical devices.

## 2. Liquid Crystalline Solutions Studied by Nuclear Magnetic Resonance (NMR) Methods

### 2.1. Liquid Crystalline Cellulose Solutions Flow in Capillaries

**Figure 4:**
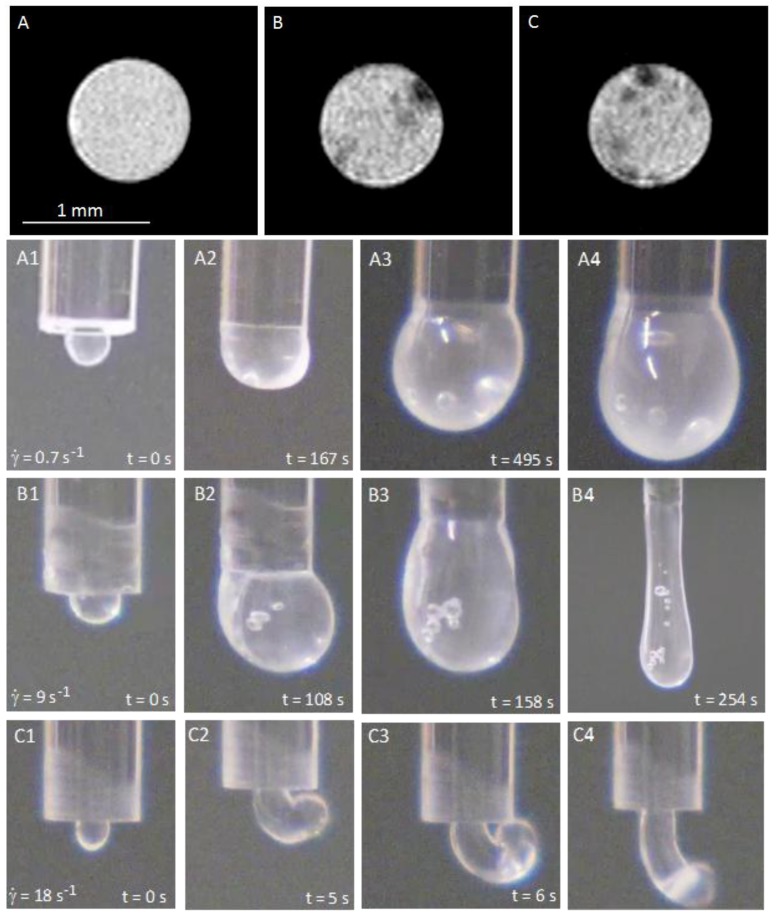
Cellulosic solutions in a straight glass capillary. (**A**–**C**) are MRI images of the cross-section of a capillary filled with: (**A**) an isotropic cellulosic solution (20% w/w, APC/DMac); (**B**,**C**) a liquid crystalline solution (60% w/w, APC/DMac) in subsequent cross-sections. Solutions were continuously sheared for 10 min (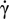 = 20 s^−1^) and frozen before MRI measurements. (**A1**), (**B1**) and (**C1**) show the time sequence illustrating the jet escaping of the free capillary end generated by different shear rates (in all three cases for the liquid crystalline solution). Reproduced from [33] with permission from The Royal Society of Chemistry.

In order to understand the origin of the intrinsic curvature observed for cellulosic liquid crystalline materials, Magnetic Resonance Imaging (MRI) experiments were performed on different acetoxypropylcellulose (APC) dimethylacetamide (DMac) solutions. The synthesis of APC was performed according to the procedure previously described in [34]. The MRI data were taken on a vertical wide bore 7.05 T Bruker 300 AVANCE III NMR spectrometer (Rheinstetten, Germany) according to [33]. The studies were performed on cellulosic solutions that were sheared in capillaries with different diameters. The flow inside the capillaries and the jet at the end of them were observed. Depending on the shear rate and on the anisotropic characteristics of cellulosic solutions, the jet showed spontaneous curvature and torsion. MRI analysis allowed imaging of characteristic structure at chosen filament cross-sections along the capillary tube as can be observed in Figure 4.

**Figure 5:**
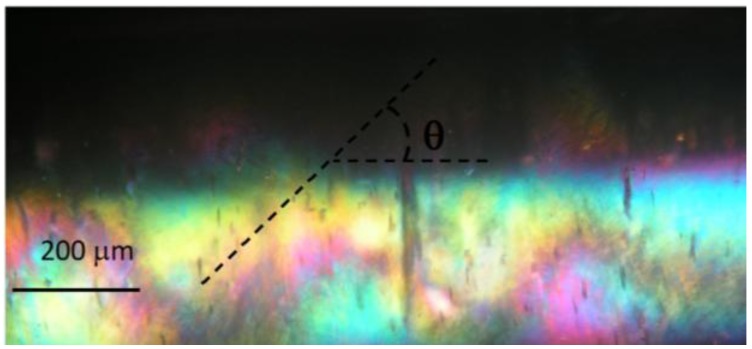
A representative POM image, under cross polarizers, of an APC/ dimethylacetamide helical liquid crystalline precursor solution inside a glass capillary (600 µm). The sample was frozen after being sheared (shear rate; 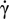 = 18 s^−1^) for 10 min. The axis of the tube is parallel to one of the polarizers. The helix radius, *R*, pitch *P* = 2π*R*/tanθ and the helical angle θ are marked on the image. The natural curvature and twist parameters are set by *k* = 1/*R* and τ = *P*/2π*R*^2^, respectively. Reproduced with permission from [33]. Copyright 2010, The Royal Society of Chemistry.

Isotropic solutions confined in the capillary, which generate straight fibers on extrusion, showed a homogeneous symmetric cross-section structure (Figure 4A), implying that the averaging of differing structural features would maintain a straight fiber conformation. Confined solutions of cellulose in the cholesteric liquid-crystal phase, which generate curved fibers, showed a heterogeneous structure in cross-section with the hard “island” predominantly located closest to the tube walls and never in the middle of the tube. The off-axis position of the hard “islands” varies along the tube (Figure 4B,C). POM confirmed that the hard “islands” correspond to the core of a linear topological defect (disclination) that can be observed in anisotropic pre- sheared solutions above a certain critical shear rate (Figure 5). POM images show that the defects lines are forming a helix along the capillary tube. This observation is supported by the MRI measurements indicates that from one layer to the following the hard objects rotate (in Figure 4 an anticlockwise rotation can be seen). The MRI image contrast reflects the local mechanical stiffness of the material, so that dark regions represent hard “islands” on each cross-section, with the average diameter from 25 to 50 μm, which is much lower than the capillary diameter (R*) and similar to the average diameter of the helices measured in POM images.

The present results show that liquid crystalline disclinations are at the origin of the intrinsic curvature observed for jets and electrospun fibers obtained from cellulosic mesophases. The intrinsic curvature of the fibers, responsible for their curl and twist, arises from an off-axis core line defect disclination which is present when the fibers were prepared from liquid crystalline solutions. The consequence of the intrinsic curvature of the system was also observed during thinning and break-up of jets produced by continuous motion of anisotropic solutions. The spirals and helices found at micro/nano scale mimic the shapes observed, for example, in hair [35] and tendrils [36] due to a similar intrinsic curvature dominated mechanism, providing that, for all these systems, the physical model proposed for elastic rods with intrinsic curvature could be applicable. The fine-tuning of temperature will allow us to control the non-woven self-winding mats’ ability adjusting their surface-to-volume ratio, due to the intrinsic curvature of constituent fibers, which opens horizons for fabricating new natural scaffolds for tissue engineering [33].

### 2.2. Rheo-NMR Studies of Precursor Cellulosic Solutions

The characterization of precursor cellulosic solutions was found to be crucial to understand the relation between properties and structure. In order to perform this goal, Rheo-NMR techniques have been used in the study of liquid crystalline cellulosic solutions under shear [37] and relaxation [38] while NMR spectroscopy was applied to the study of the corresponding films and fibers [39]. These studies allowed for the characterization of the molecular order and structure in these systems, yielding information not directly accessible to other techniques as light scattering [40] or Xray diffraction [41]. 

**Figure 6:**
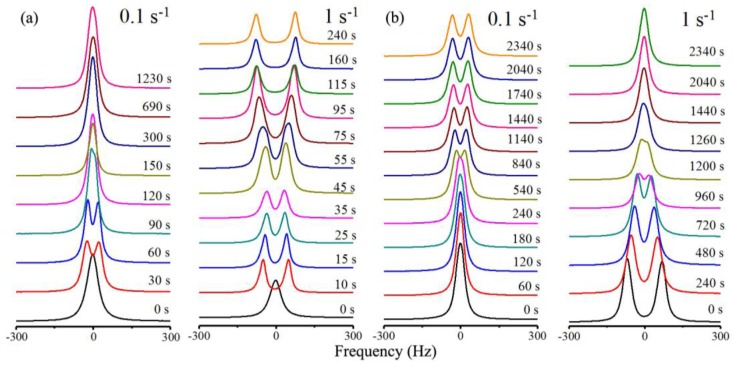
NMR spectra evolution of HPC in D_2_O. (**a**) During the shearing process with shear rates of 0.1 and 1 s^−1^; (**b**) after the cessation of shear. Reprinted with permission from [42]. Copyright 2013, American Chemical Society.

A Rheo-NMR study [42,43] on the model system hydroxypropylcellulose + deuterium oxide (HPC + D_2_O) at 50% by weight concentration showed a rich and complex behavior both under shear and in relaxation as can be observed from the deuterium spectra recorded in both processes and shown in Figure 6 for selected shear rates.

It was found under shear that depending upon the shear rate the system goes from a regime of tumbling cholesteric domains [44,45] observed at low shear rates 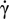 = 0.1 s^−1^ where the system order is low, to a regime of director flow alignment with partial or total helix suppression for shear rates 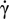 of the order of 1 s^−1^ or above. The three distinct flow regimes introduced by Onogi and Asada [46] in the context of shear flow of LC polymers were directly identified in the Rheo-NMR [47] results and were ascribed to the tumbling of cholesteric domains, the partial flow alignment of the director and helix deformation and the director flow alignment with partial or total helix suppression. Relaxation data in the different regimes show that the system moves towards the reformation of the cholesteric domains and depending upon the starting point where the system is left in to relax this process may be more or less involved. When relaxation initiates from the tumbling cholesteric domains regime the cholesteric domains grow and possibly accrete giving rise to an immediate increase in order. When relaxation initiates from the flow director aligning regime, cholesteric helices start to form from the aligned director state, producing a significant decrease in order that only reverts when the reformed cholesteric domains start to accrete and grow, a process that takes significantly longer times to develop. 

**Figure 7:**
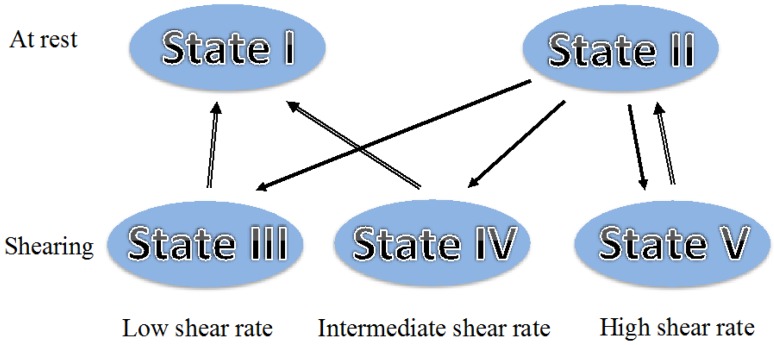
Mesoscopic states reached by the HPC/water system during shear and relaxation. Reprinted with permission from [42]. Copyright 2013, American Chemical Society.

This study was carried out through the analysis of the deuterium spectra arising from the D_2_O molecules and shown in part in Figure 6. The spectra showed the presence of a single population of D_2_O molecules experiencing a large motional averaging. The deuterium spectra were analysed and simulated considering the presence of water rich regions (WRR) imbedded with the polymer chains and the presence of fast molecular diffusion within these WRR that allow the D_2_O molecules to sample the order of the neighbouring polymer chains. Based on those results, and the accumulated knowledge on this system [37], it was possible to identify five different mesoscopic states reached by the HPC/water system during shear and relaxation as shown in Figure 7. State I is the partially aligned cholesteric polydomain state obtained after relaxation from shearing with low shear rates. State II is the cholesteric polydomain state attained after relaxation from shearing with high shear rates. State III is the tumbling regime of the cholesteric domains obtained with low shear rates, state IV is the partial flow alignment state associated with the intermediate shear rates, and state V is the flow aligned nematic obtained at high shear rates. The up arrows indicate the relaxation path while the down arrows indicate the path under shear starting from the polydomain cholesteric reached in the preparation state. This picture is in direct correspondence with the three regions of steady shear flow introduced by Onogi and Asada [46], state III appears at low shear rates and corresponds to the tumbling regime, the intermediate region corresponds to state IV and state V is associated with the high shear rate region.

## 3. Liquid Crystalline Cellulose Networks Characterized by Different Experimental Techniques

### 3.1. Solid State NMR Spectroscopy of APC Networks With Nematic 5CB Inclusions

**Figure 8:**
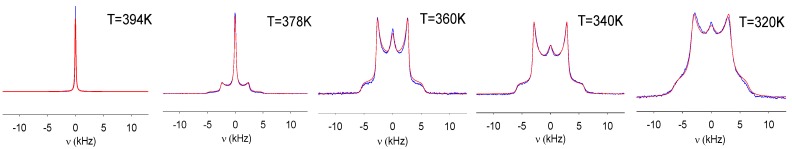
Deuterium spectra obtained in spun fibers at different temperatures starting within the isotropic phase (**left**) and going deeply inside the nematic range (**right**) (blue line, data; red line, model fit). Reprinted with permission from [39]. Copyright 2010, American Chemical Society.

In a recent NMR study [39], bulk samples, thin films and electro spun fibers of the cellulosic composite formed by liquid crystalline acetoxypropylcellulose (APC) and deuterated nematic 40-pentyl-4-cyanobiphenyl (5CB-αd2) with the percentage of 85% APC by weight were characterized by deuterium spectroscopy in terms of molecular orientational order. Deuterium spectra obtained in thin films and bulk samples show similar temperature dependences as the one found in spun fibers and shown in Figure 8.

It was found that the low molecular weight liquid crystal is phase separated from the APC and concentrates on submicron droplets in the three types of samples. These droplets are seen to develop a nematic wetting layer at the APC-5CBαd2 interface that experiences an order-disorder transition driven by the polymer network N-I transition. Simulation of the deuterium powder patterns as a function of temperature has shown that the APC network I-N transition exhibits a pronounced subcritical behavior within a heterogeneity scenario.

**Figure 9:**
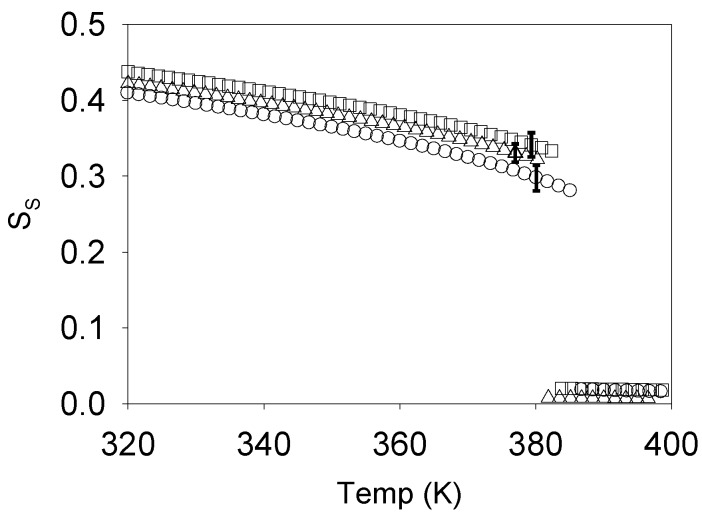
Temperature dependence of the order parameter S_S_ for *T*_Ci_ = <*T*_C_> for the three distinct samples studied. Triangles refer to the fiber sample, squares to the bulk sample and circles to the film sample. Reprinted with permission from [39]. Copyright 2010, American Chemical Society.

This study relied on the analysis of the deuterium spectra arising from the 5CBαd2 molecules as they diffuse inside the submicron size droplets, sampling both their isotropic core, as well as the polymer surface induced nematic layer. A Landau-de Gennes modelling of this induced order allowed the order profile inside the droplets to be determined as a function of the surface order S_S_ imposed by the polymer chains, enabling the simulation of the ordered part of the deuterium spectra shown in Figure 7. At the network I-N transition a significant coexistence of both isotropic and ordered components in the deuterium spectra indicates the presence of heterogeneity in the network. To model the network order parameter temperature dependence and the network heterogeneity, a Landau de Gennes modeling of the network order was considered with a Gaussian distribution in the isotropic phase super cooling limit temperature T* [39]. This modeling allowed the full fitting of the spectra shown in Figure 8 and the determination of the S_S_ temperature dependence shown in Figure 9.

The S_S_ temperature dependence shows that the network is subcritical and the spread in transition temperatures of around 7 K for each of the three samples studied indicates the presence of heterogeneity in the APC + 5CBαd2 system [39].

### 3.2. Wide Angle X-ray Scattering and Polarized Optical Microscopy of Crosslinked APC Films under Strain

**Figure 10:**
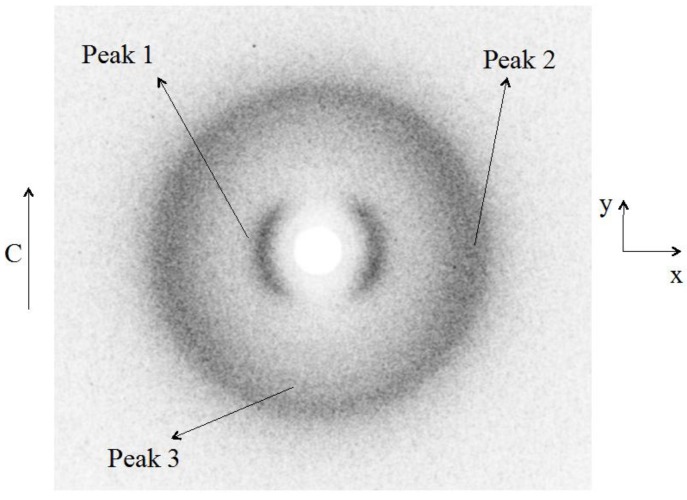
X-ray diffraction pattern of the unstretched cellulose film. The single arrow represents the casting direction. Peaks 1, 2, and 3 are identified. Reprinted with permission from [29]. Copyright 2011, Springer.

**Figure 11:**
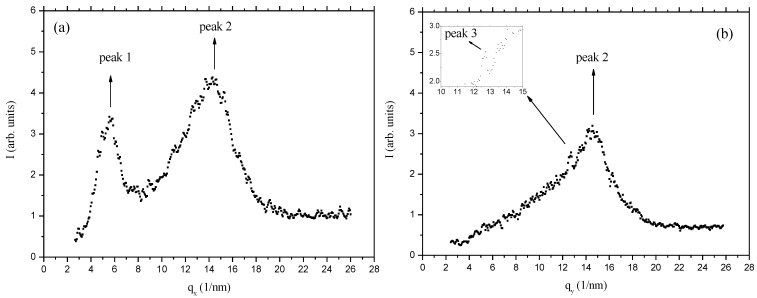
X-ray diffracted intensity of unstretched cellulose film. (**a**) As a function of q, along the x-axis; (**b**) as a function of q, along the y-axis. The insert refers to a zoom around q ≈ 13 nm^−1^. Reprinted with permission from [29]. Copyright 2011, Springer.

Cellulosic liquid crystalline networks obtained from the previous studied systems can be at the origin of promising soft matter devices [24]. In order to characterize these type of networks, Wide Angle X-ray Scattering (WAXS) studies were performed [48] namely of crosslinked APC films produced from chiral nematic solutions [29]. The studies were carried on films subjected or not to a uniaxial stress. The results indicate that the films are constituted by a bundle of helicoidal fiber-like structures, where the cellobiose block spins around the axis of the fiber. Without the stretch, these bundles are warped, only with a residual orientation along the casting direction. The stretch orients the bundles along it, increasing the nematic-like ordering of the fibers. Under stress, the network of molecules that connects the cellobiose blocks and forms the cellulosic matrix tends to organize their links in a hexagonal-like structure. The X-ray diffraction patterns of the free standing, unstretched, cellulose films present three diffraction peaks, anisotropically disposed around the z-axis, as shown in Figure 10. In Figure 11a,b, the diffracted intensity is plotted as a function of q (scattering vector), along the x and y axes directions. The diffraction peaks in the curves Ixq were fitted with Lorentzian functions to find the characteristic distances (d) and full-widths at half- height (W).

The characteristic distances associated to the peaks are *d*_1_ = (1.13 ± 0.02) nm; *d*_2_ = (0.44 ± 0.01) nm; and *d*_3_ = (0.50 ± 0.01) nm. In a previous work [30] only peak 1 was observed due to the limited range of scattering wave vectors investigated. The correlation lengths *D* = λ_x_/(*W*·cosθ) calculated are *D*_1_ ≈ 3 nm; *D*_2_ ≈ 2 nm; and *D*_3_ ≈ 14 nm.

The analysis of the diffraction peaks in the x–y plane indicates that peaks 1 and 2 are more intense along the x direction, which corresponds to the direction perpendicular to the casting. On the other hand, we could not fully verify if peak 3 is also anisotropic with respect to the z-axis since the presence of peak 2, particularly along the x-axis direction, prevents a reliable azimuthal analysis of it. Nevertheless, visual inspection of Figure 10 seems to indicate that this peak is preferentially located in the direction perpendicular to the casting direction. The azimuthal angle analysis of the diffracted intensity (angle φ, measured in the xy plane, starting from the x-axis, in the counter clockwise direction) of the peaks allowed us to obtain the orientational order parameters (OP) [29].

**Figure 12:**
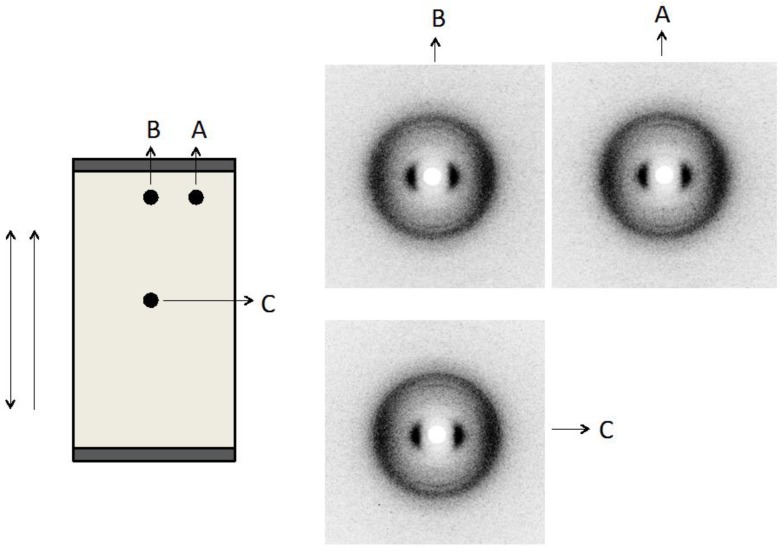
Cellulose film at maximum stretch with ɛ_y_ = 0.40. (**a**) Sketch of the cellulose film under stretch and indication from where the X-ray diffraction patterns were taken. The single arrow indicates the casting direction and the double arrow the stretching direction; (**b**) diffraction patterns obtained at the different film location. Reprinted with permission from [29]. Copyright 2011, Springer.

Concerning the case where the film is subjected to a uniaxial stress parallel to the casting direction (Figure 12). We did not observe significant differences in the patterns obtained in the different film locations of the X-ray exposures, indicating, at least, in our experimental conditions, homogeneity of the film.

**Figure 13:**
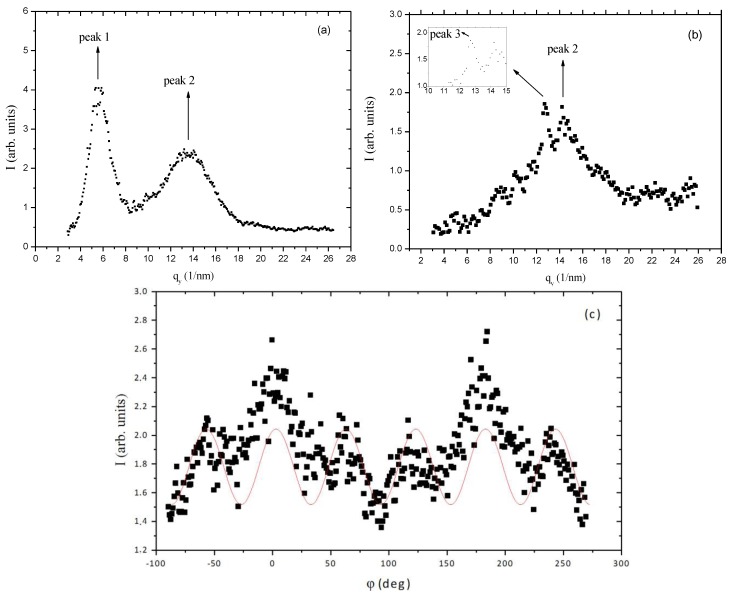
X-ray diffracted intensity of stretched cellulose film at maximum stretch with ɛ_y_ = 0.40. (**a**) As a function of q, along the x-axis; (**b**) as a function of q, along the y-axis. The insert refers to a zoom around *q* ≈ 13 nm^−1^; (**c**) as a function of the azimuthal angle φ, referring to peak 2. The sinusoidal curve is only a guide for the eyes. Reprinted with permission from [29]. Copyright 2011, Springer.

As the stress increases, the order parameters of the three peaks increase, reaching values at the maximum stress applied (ɛ_y_ = 0.40) of OP_1_ = (0.85 ± 0.03); OP_2_ = (0.43 ± 0.02); and OP_3_ = (0.82 ± 0.01). This increase in the orientational order parameter of peak 1 is consistent with the results obtained in [30]. Figure 13 shows the diffracted intensity as a function of q along the x (Figure 13a) and y (Figure 13b) axis directions. As the stretch is increased, peak 3 become more and more defined in the diffracted intensity *versus* q curves (Figure 13b). The positions of the peaks were not modified by the stress. An intriguing result was obtained when we analyzed peak 2 profile as a function of the azimuthal angle φ (Figure 13c). 

A hexagonal distribution of diffraction maxima could be identified in the peak 2 position, which provided a hexagonal lattice parameter *d*_H_ ≈ 0.5 nm about the same of the characteristic distance d_3_. The value of d_H_ was obtained assuming that the diffraction peaks of hexagonal symmetry observed in the position of peak 2 (*d* ≈ 0.44 nm) correspond to the (100) diffraction plane of the two-dimensional hexagonal packing, *i.e*., *d*_H_ = *d*_100_/(cos(π/6)). The typical dimension of an extended glucose molecule is of the order of 0.5 nm and the cellobiose, composed by two glucose molecules, is the repeating building-block in the cellulosic sample. The structure proposed for the cellulosic film has to take into account the diffraction characteristic distances observed, the dimension of the cellobiose building block, and the information that, before the casting, the system presents a cholesteric structure. We propose that in the film there is a bundle of helicoidal fiber-like structures where the cellobiose block spins around the axis of the fiber. The distance between the fibers should be of the order 1.1 nm, corresponding to peak 1 of the diffraction pattern. Since there is no evidence of chiral activity in the cellulosic films in the macroscopic scale, these bundles should have fibers with both the levogyre and dextrogyre arrangements, with equal probabilities. Without the stretch, these bundles of fibers may be warped, only with a residual orientation along the casting direction. The stretch orients the bundles along it, increasing the nematic-like ordering of the fibers. Under stress, the network of molecules that connects the cellobiose blocs and forms the cellulosic matrix tends to organize their links in a hexagonal- like structure.

**Figure 14:**
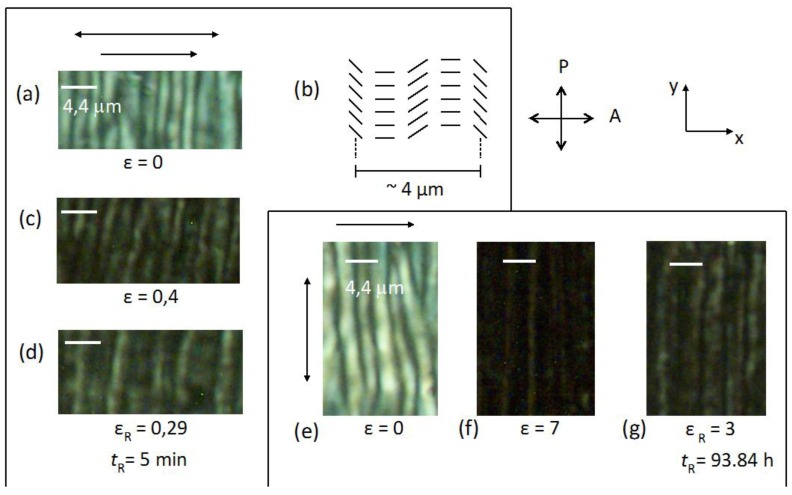
Optical microscopic textures under crossed polarizers (OMP) of (**a,e**) the film unstretched; (**b**) and sketch of the director orientation; (**c**) OMP of the film under stretch parallel to the casting direction; and (**d**) upon relaxation; (**f**) OMP of the film under stretch perpendicular to the casting direction; and (**g**) upon relaxation. The single arrow indicates the casting direction and the double arrow the stretching direction. Reprinted with permission from [29]. Copyright 2011, Springer.

The same samples used to perform the WAXS essays were used to perform POM essays. The optical microscopy texture of the sample under crossed polarizers (casting direction parallel to the analyser) revealed that the film is birefringent, but with a non-uniform texture (Figure 14a).

The analysis of the texture under crossed polarizers leads to the macroscopic arrangement of the director sketched in Figure 14b. There are light and dark regions that form a pattern with stripes, with a periodicity of ≈ 4.4 μm (distance between three light regions), perpendicular to the direction of the casting. This periodic structure is originated by the shear-casting procedure. After the cast the molecular chains have a collective relaxation that results in the formation of that pattern. This morphology has been observed in other cellulose derivative films and was found to be influenced by the precursor solution composition, solvent evaporation rate, film thickness, and rate and duration of shear [28,49]. The application of successive stretches in the direction of the casting (*i.e*., in this experimental configuration, parallel to the analyser direction) showed that the texture of the film becomes increasingly dark. This is because the direction of the optical axis, previously created by the casting, is parallel to the analyser direction. If the optical axis of the sample is rotated by 45° in the plane of the microscope plate, the texture became bright. This result is consistent with the nanoscopic structure proposed in the previous X-ray scattering section. The fiber-like structure where the cellobiose block spins around the axis of the fiber defines the director direction, parallel to the casting direction. As the X-ray beam probes a large portion of the sample (typical beam diameter of about 1 mm) the diffraction pattern reveals the mean orientation direction of the director. Upon relaxation (initially at ɛ = 0.29 and after *t*_R_ = 5 min) the film did not recover the initial texture, in accordance with the X-ray results already discussed.

**Figure 15:**
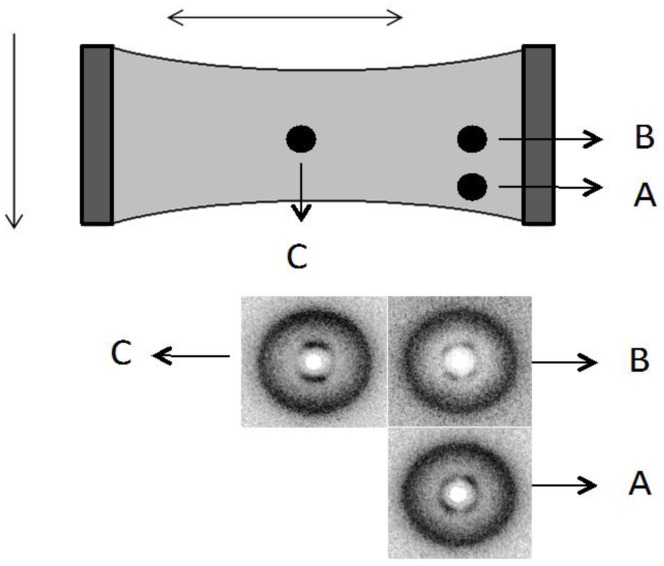
Sketch of the cellulose film under stretch (ɛ = 7.00) and indication from where the X-ray diffraction patterns were taken. The single arrow indicates the casting direction and the double arrow the stretching direction. Diffraction patterns obtained at the different film locations. Reprinted with permission from [29]. Copyright 2011, Springer.

When the stretch is applied perpendicular to the casting direction the texture of the sample under crossed polarizers (Figure 14e,f) becomes increasingly darker with increasing stretch, and then clearer after its relaxation (Figure 14g). Assuming the structure depicted in Figure 14b, the stretch along the direction perpendicular to the casting will impose a tendency of reorientation of the fiber-like arrangement parallel to the stretching direction. This reorientation may in principle proceed either in the clockwise or the anticlockwise direction but due to the initial oscillatory form of the director around the casting direction due to the band structure, different regions will rotate in opposite sense depending on the initial director orientation at each point. Depending on the location of the film analyzed and the boundary conditions imposed by the borders of the film, the fibers will tend to reorient to their local environment. This fact can explain the different X-ray patterns obtained in different film positions (Figure 15). At the border of the film (positions A and B in Figure 15) the director, that was initially oriented on average parallel to the casting direction (x-axis), is now primarily oriented at ± π/4 with respect to the x-axis due to the boundary conditions that prevent a complete rotation of the director to the stretching direction. In the center of the film (position C in Figure 15), the director is oriented primarily along the y-axis (stretching direction). Let us look in more detail at the appearance of the film under stress, without the crossed polarizers. The film initially had 2 mm in length (L_0_), 5 mm in width and 21 μm in thick and was stretched every 6 min until 16 mm in length, and then relaxed. 

**Figure 16:**
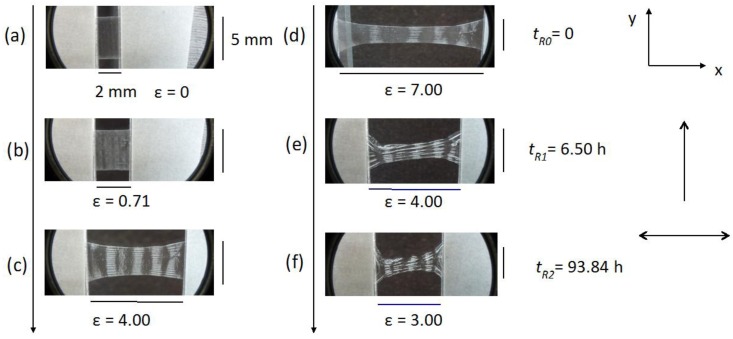
Textures from optical microscopy without crossed polarizers (OM) of the film unstretched (**a**); under successive stretches (**b,c,d**); and under relaxation (**e,f**). Stretch perpendicular to the casting direction. The single arrow indicates the casting direction and the double arrow the stretching direction. Reprinted with permission from [29]. Copyright 2011, Springer.

Figure 16 shows the sequence of stretches of the film from ɛ = 0.71 (Figure 16b) until ɛ = 7 (Figure 16d), with the stress applied perpendicular to the casting direction. It is clearly observed an additional periodicity in the direction perpendicular to the casting direction. 

**Figure 17:**
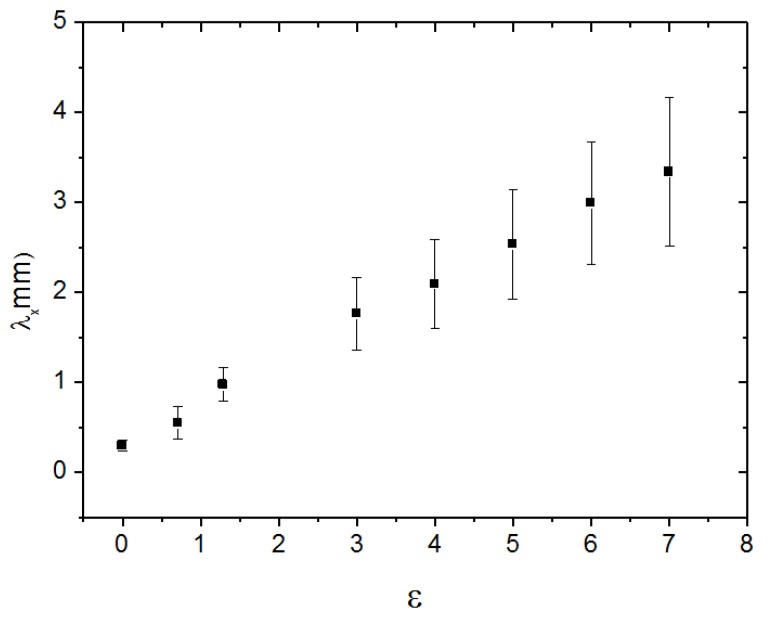
Distance between two consecutive stripes along the x-axis, as a function of ɛ. Data corresponding to the experimental situation of Figure 16. Reprinted with permission from [29]. Copyright 2011, Springer.

**Figure 18:**
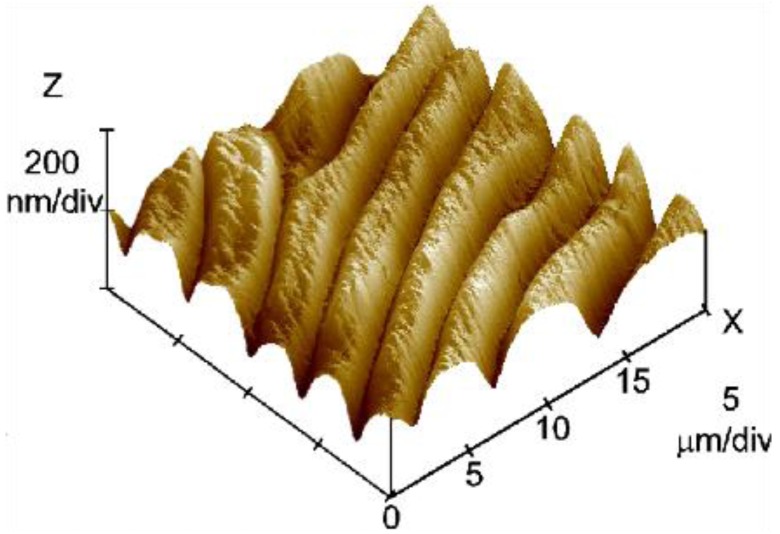
3D topography image (20 × 20 μm^2^ scan) of the free surface of a sheared HPC film prepared from a 60% w/w solution at a shear rate *v*_1_ = 5 mm/s. Reprinted with permission from [28]. Copyright 2002, American Chemical Society.

The distance (along the x-axis) between two successive stripes increases linearly with ɛ (Figure 17). Under crossed polarizers these stripes are also birefringent (Figure 18). This result indicates that the effect of the casting in the macroscopic structure of the cellulosic film is not only to impose a periodic bend organization of the local director, as sketched in Figure 14b in its direction, but also bends this super structure in the direction perpendicular to it in a larger length scale. Upon relaxation, the film takes a long time to recover its original length (Figure 16e,f). However, its shape is strongly modified, in particular its width (direction perpendicular to the stretch), indicating the plastic behavior of the deformation.

When the film is stretched in the direction parallel to the casting direction this additional periodicity is not observed.

### 3.3. Atomic Force Microscopy of HPC Films

To perform the Atomic Force Microscopy (AFM) experiments, solutions of cellulose derivatives at several concentrations, ranging from the isotropic to the anisotropic phase region, were prepared. 

Figure 18 shows the 3D topography image (20 × 20 μm^2^ scan) of the free surface of a sheared HPC film prepared from a 60% w/w solution at a shear rate *v*_1_ = 5 mm/s. The image shows two different scale ranges: a primary set of “large” bands, perpendicular to the shear direction, and a smoother texture characterized by a secondary periodic structure containing “small” bands.

**Figure 19:**
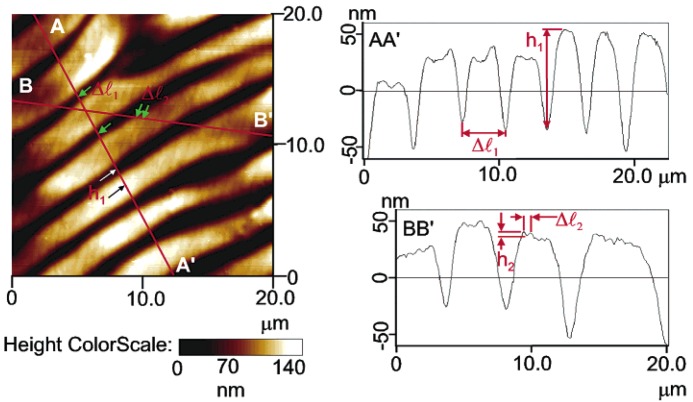
Top view image of the height scan of the surface shown in Figure 20 and the height profile analysis at the two cross sections: AA' and BB'. The arrows on the top of a view image along AA' and BB' lines mark the points used for the measurements of the height profile. Reprinted with permission from [28]. Copyright 2002, American Chemical Society.

Figure 19 shows a top view image of the height scan of the surface shown in Figure 18 and the analysis of the height profile at two cross sections: AA' and BB'. Cross section AA' was taken along the shear direction. The periodicity of the “large” bands, ∆*l*_1_, and the average peak-to-valley height for these bands, *h*_1_, were determined from the AA' height profile plot, as indicated. Cross section BB' was taken along the direction of the secondary periodic “small” bands. The periodicity of the “small” bands, ∆*l*_2_, and their peak-to-valley height, *h*_2_, were measured from the BB′ height profile plot, as indicated. The arrows on the top of the view image along AA' and BB' lines mark the points used for the measurements performed in the height profile plots.

**Figure 20:**
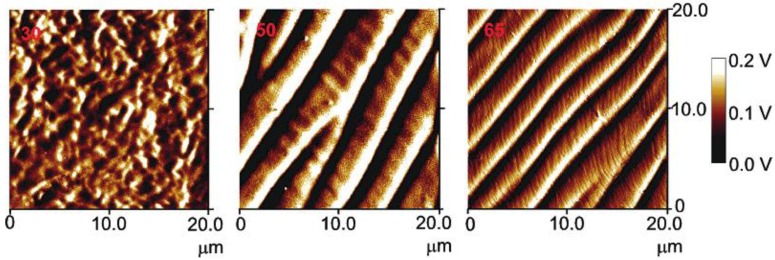
Top view image of the amplitude scan of the free surface of three sheared HPC films prepared at a shear rate of *v*_1_ = 5 mm/s from solutions with HPC/water ratios: (**a**) 30% (w/w); (**b**) 50% (w/w); and (**c**) 65% (w/w). Reprinted with permission from [28]. Copyright 2002, American Chemical Society.

Figure 20 shows the top view image of the amplitude scan of the free surface of three sheared HPC films prepared at a shear rate of *v*_1_ = 5 mm/s from solutions of different concentrations: (a) 30% (w/w), (b) 50% (w/w); and (c) 65% (w/w). The surface of the film prepared from 30% (w/w) does not possess any periodicity. A primary and a secondary set of bands were observed only on the films prepared from anisotropic solutions, *i.e*., 50%–65% (w/w). Moreover, the films prepared from the solutions of the same concentration, using a higher shear rate v_2_ = 10 mm/s), exhibit similar topographies, but they are characterized by different parameters. At a constant concentration, for example at 65% (w/w), the periodicity of the bands (∆*l*_1_) shows a tendency to decrease when the shear rate increases (∆*l*_1_(*v*_1_) = 2.97 μm and ∆*l*_1_(*v*_2_) = 1.97 μm). At a constant shear rate, as the concentration of the polymer increases, the periodicity of the bands decreases (for *v*_1_, at 50% and 65% (w/w), ∆*l*_1_ = 4.68 μm and ∆*l*_1_ = 2.97 μm, respectively).

A tuneable topographic system may be obtained from HPC aqueous liquid crystalline solutions [50]. The results point out that two kinds of periodicities may be locked and adjusted in these systems as a function of the processing conditions. The set of “large” bands, which develops perpendicular to the shear direction, can be described as a relaxation process, which occurs immediately after the end of a shear applied to polymer liquid-crystalline solutions and attributed to contraction strains of the sheared sample induced by stress relaxation after cessation of flow. The secondary bands periodicities show a net tendency to decrease with polymer content, which seems an indication that the development of the “small” bands are mainly ruled by the cholesteric liquid crystal characteristics imposed by the initial precursor solutions. The pitch of the precursor chiral nematic solution and the related values of the liquid crystal elastic constants of the material can therefore be mainly responsible for the variation in size of the secondary bands, observed experimentally [24]. The films are found to be self-affine between 300 nm and 4 μm but not for higher scales. In general, the fractal dimension is found to increase with both polymer concentration and shear rate. This trend reflects the increasing complexity of the surface topography when the films are prepared with higher polymer concentrations or with higher shear rates [28].

### 3.4. Mechanical Behavior of Solid Cellulose Derivatives Films

Anisotropic solid films were fabricated by spreading the HPC anisotropic solutions, 60% (w/w), with the help of a calibrated shear casting knife, at room temperature, in an appropriate Teflon mould, this procedure allows the precise control of the shear casting flow speed (*v*) (1 mm·s^−1^) and enables the ready removal of the films without damaging at room temperature, other methods to obtain sheared HPC solid films involved higher processing temperatures [51]. After solvent controlled evaporation, the solid cast shear films had an average thickness between 14 and 30 μm. As evaporation continues, the density of rod-like fragments increases near the free top surface, giving rise to increased orientational order. This in turn causes elongation at the top of the film in the direction parallel to the director, and since the dimensions of the bottom surface in contact with the glass substrate are fixed, the top surface buckles, forming a set of grooves shown previously [52,53,54].

**Figure 21:**
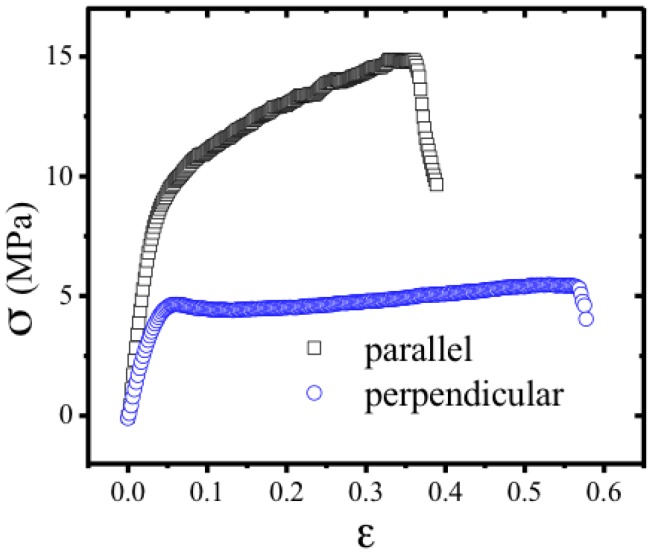
Stress-strain relations. Squares correspond to strain parallel to the shear direction and the nematic director. Circles correspond to strain perpendicular to the shear direction and the nematic director. For this geometry, above a threshold, the stress is nearly independent of strain. This indicates “semi-soft” elasticity, characteristic of nematic elastomers. The negative slope at higher strains corresponds to failure due to tearing of the films. Reprinted with permission from [55]. Copyright 2013, Nature Publishing Group.

The modulus of the cellulose network has been measured for strain along the director, as well as perpendicular to it. Results are shown in Figure 21; for small strains, Young’s modulus is 263 ± 39 MPa for shear parallel and 140 ± 9 MPa perpendicular to the director. Above a threshold of 4 MPa, the stress in the perpendicular direction is nearly independent of the strain. This “semi-soft” elastic response is characteristic of liquid crystal elastomers [56] and has been associated to a director reorientation. However, as already highlighted [57], a reset of order can also be the underlying mechanism for this process as observed in sheared anisotropic cellulosic films [30]. The film rupture is preceded of a plastic regime when stretching is imposed parallel to the casting direction while it is preceded of significant necking during the “semi-soft” elastic response when stretching is imposed perpendicular to the casting direction.

**Figure 22:**
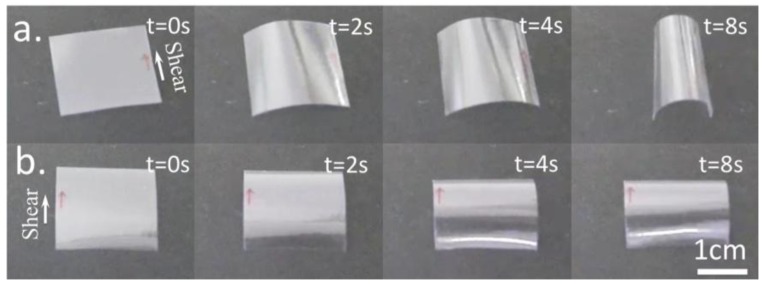
Bending of freestanding films. (**a**) The film free top surface; and (**b**) the film glass bottom surface exposed to water vapour. Sheared films were prepared from liquid crystalline HPC/water solution, the arrows indicate shear direction. Reprinted with permission from [55]. Copyright 2013, Nature Publishing Group.

When exposed to water vapor, free standing films prepared from a 60% (w/w) solution bend, as shown in Figure 22. When water vapor penetrates the free surface of the film, the sample bends around an axis parallel to the shear direction, with the free surface on the outside (Figure 22a). This is consistent with expansion of the free surface of the film in the direction perpendicular to the director. Such an expansion is expected, since the order parameter is reduced by the presence of the solvent water, and furthermore the presence of water molecules between the cellulose chains in the rigid segments is expected to cause the thickness of the rod-like fragments to increase.

**Figure 23:**
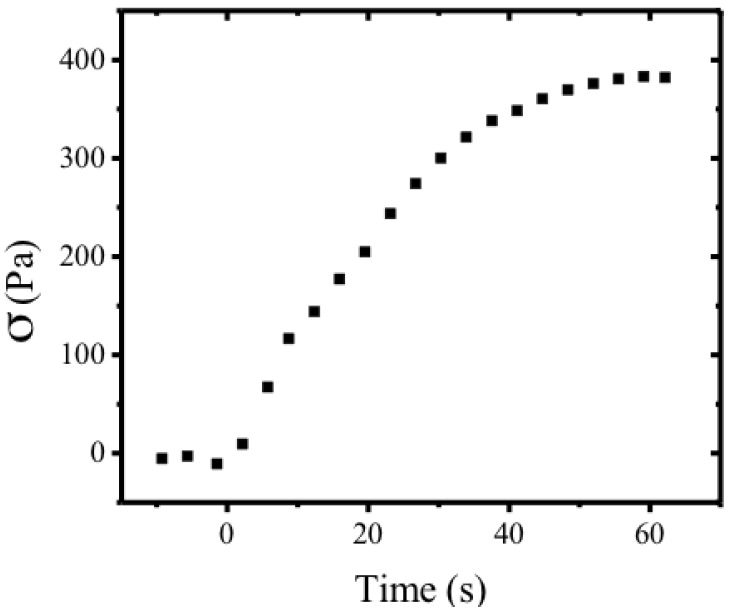
Dynamics of stress evolution. Shear stress in a 20 mm × 20 mm × 32 µm planar sample at 24 °C with free surface exposed to humidity as function of time, measured with a Mettler Toledo AG204 load sensor. The maximum stress measured was 383 Pa. Reprinted with permission from [55]. Copyright 2013, Nature Publishing Group.

The shear stress associated with such bend has been measured, as a function of time (Figure 23), in a 20 mm × 20 mm × 32 μm planar sample at 24 °C with free surface exposed to humidity. Measurements were taken with Mettler Toledo AG204 load sensor. The maximum stress measured was 383 Pa.

When the film is allowed to dry, either by heating or by being placed in a low-humidity environment, the film unbends, reversibly assuming its original shape. The bending is relatively fast, on the scale of ≈1 s.

**Figure 24:**
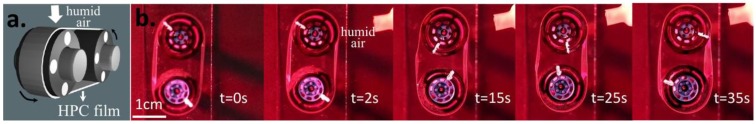
Moisture-driven liquid crystal cellulose engine. (**a**) Schematic of the motor, showing location of moist air and rotation direction. The alignment direction is parallel to the axes of the wheels. The free surface of the film is on the outer side; (**b**) series of video frames showing rotation. The motor is housed in a dry environment. Momentum transfer from the moist air is small, and opposes the observed motion. Belt dimensions are: 1.0 cm × 8.0 cm × 30 µm; wheel diameter is 14 mm. The direction of rotation is indicated by a black arrow, and of moist air flow by a white arrow (**a**). In (**b**), t = 2 s/frame. Reprinted with permission from [55]. Copyright 2013, Nature Publishing Group.

Interestingly, when the glass side of the film is exposed to water vapor, it bends around an axis perpendicular to the shear direction, with the glass side being convex (Figure 22b). This is consistent with a nearly isotropic expansion of the glass side surface due to the presence of moisture. Since corrugations on the free surface give rise to a smaller effective modulus for bend in this direction, as has been confirmed by independent measurements, bend occurs around an axis perpendicular to the shear direction. The bend produced by exposing the glass side to water vapor is considerably smaller than that of the free surface. Taking advantage to the bending moisture effect a soft cellulosic motor was built (Figure 24) [55].

Nano crystalline cellulose rods can be incorporated into composite materials, enhancing their mechanical properties [58]. NCC filler was used as a probe, which can influence the mechanical properties of the films but does not destroy the liquid crystalline characteristics of the composite material. In fact, adding 0.1% of NCC rods implies that the Young’s modulus of the films, as well as the tensile strength, measured in perpendicular (Per) and parallel (Par) directions to the casting, were increased by a factor of 2.5 and 3.2 for Par and 3.0 and 2.2 for Per, respectively, when compared with films prepared from HPC anisotropic solutions [24]. Because of the high degree of molecular orientation, the HPC and the HPC/NCC films exhibit high modulus and strength along the shear direction and the mechanical strength in the transverse direction is low. These anisotropic mechanical properties are consistent with the molecular orientation, which results from the flow of the liquid crystalline solution under shear stress. The fact that the Young’s modulus and strength is much higher for HPC/NCC compared with HPC films, along the shear direction, is an indication that NCC rods align along this direction when the films are prepared. NCC rods enhanced the brittle behavior along this direction, as well as in the Per direction and act as a stiffener to the anisotropic cellulose matrix, which is also reflected in the strength deformation values for Par and Per directions.

The features (not the values) of the stress/strain curves obtained were similar to those from other APC and HPC already reported in the literature for isotropic and anisotropic cellulose derivatives [59,60,61].

### 3.5. Cellulose Derivatives Composites in Electro-Optical Applications

**Figure 25:**
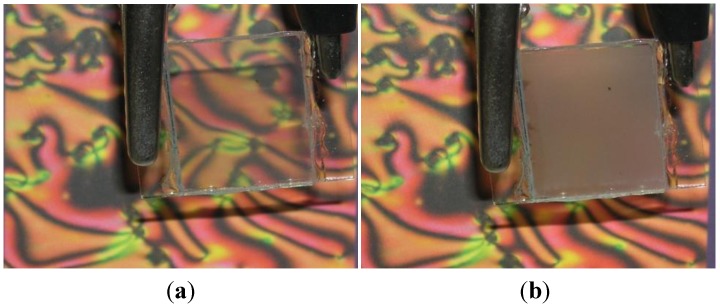
Macroscopic effect of the (**a**) ON; and (**b**) OFF states. Reprinted with permission from [66]. Copyright 2009, AIP Publishing.

Cellulose derivatives composites for electro-optical (EO) applications were introduced in 1982 by Craighead *et al*. [62], followed a few years later by a different type of cellulose derivative EO cell, named cellulose-based polymer dispersed liquid crystal (CPDLC) [63,64]. Due to the good match between the ordinary refractive index of the nematic liquid crystal (NLC) E7 (1.510) and the refractive indexes of HPC (1.49), a very clear ON state is achieved when an electric field *E*_on_ is applied to the devices as can be seen in Figure 25.

The CPDLC cell was composed of a rough cellulose derivative polymeric film surrounded by two NLC layers and the set placed in between two transparent conducting rigid or flexible substrates. These thin solid films were prepared from cellulose derivatives solutions, casted, and sheared simultaneously by moving a calibrated Gardner knife at 1.25 mm·s^−1^. The films were allowed to dry at room temperature and kept in a controlled relative humidity (20%) chamber until further use. To evaluate the EO properties of these devices, the EO characterization was carried out using a laser-equipped optical bench in association with a function generator, a voltage amplifier, and a diode detector. The laser light was perpendicular to each sample and upon crossing it was collected at the diode detector whose output was fed to an amplifier and later recorded with a digital storage scope. All measurements were performed at room temperature. These cells showed very challenging properties, presenting high transmission coefficients values (around 0.8) in the ON state, but exhibiting turn-ON fields around 1.5 V/µm, giving rise to rather high turn-on voltages [65]. Later on, a light scattering EO device where layers of two different cellulose derivatives were deposited as nonwoven nano and microfiber mats onto the conductive substrates by electrospinning, were presented [66]. These devices can be used as high efficiency light shutters or as privacy windows since they can be electrically controlled to scatter light (OFF state) or to transmit it (on state) [67]. These last devices presented an innovative method of preparing cellulose-based light scattering devices, which led to a major improvement in their EO properties and also their production cost. Using cellulose-based nano and microfibers mats as a network, EO light-scattering devices were produced as polymer-stabilized liquid crystal-type devices. In these devices, the cellulose derivatives were deposited as nonwoven nano- and microfiber mats onto the conductive substrates by electrospinning, and the cell was filled up by capillarity with a NLC. In these optical shutters, the LC is embedded with the fibers as a continuous phase, maximizing the LC/polymer surface contact and thus promoting improved EO properties. An increase in the on transparency and a marked decrease in the operating voltage of these devices were observed as the consequences of the improved interaction of the NLC with the nano and microfibers. More recently, a new type of EO device with improved EO properties was presented taking advantage of the high surface area of nanoscale cellulose whiskers [27]. 

**Figure 26:**
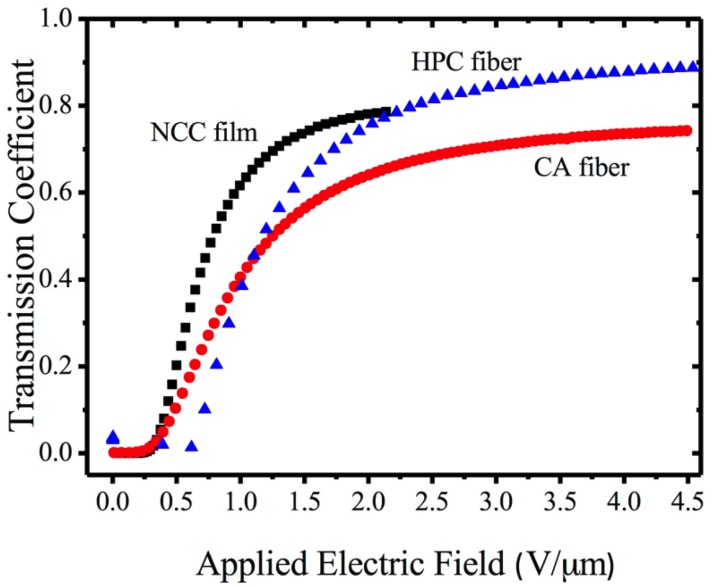
Curves of the applied electric field dependence of the light transmission coefficient of these devices compared with electrospun cellulose fiber devices (HPC and CA). Reprinted with permission from [27]. Copyright 2013, Taylor and Francis.

On this later technology, cellulose-based LC EO devices were prepared by stacking between two transparent conductive oxide–coated glasses, two layers of a nematic LC having in between a thin film composed of nano crystalline cellulose rods. The major step forward in the EO properties of this type of device is the significant reduction of the turn ON electric field and the decrease in the response time to reach the ON state as can be seen in Figure 26.

## 4. Summary

The cholesteric nature of the cellulose derivatives liquid crystalline solutions gives rise to a complex rheological behavior and consequently has a fundamental impact on the properties of the films and fibers produced from these anisotropic solutions by different methods where different levels of shear are involved. Formation of helices in electro-spun fibers and the curling of cast planar films stem from the complex nature of the molecular packing imposed by the preparation induce shear on the precursor solutions. These features impart specific mechanical and optical properties to the cellulose derivatives fibers and films that make them high interest materials for photonics, microelectronics and related applications, as well as for build soft stimuli responsive devices.

## References

[1] Werbowyj R.S., Gray D.G. (1976). Liquid crystalline structure in aqueous hydroxypropyl cellulose solutions. Mol. Cryst. Liq. Cryst..

[2] Werbowyj R.S., Gray D.G. (1984). Optical-properties of (hydroxypropyl) cellulose liquid-crystals—Cholesteric pitch and polymer concentration. Macromolecules.

[3] Kimura H., Hosino M., Nakano H. (1982). Temperature dependent pitch in cholesteric. J. Phys. Soc. Jpn..

[4] Werbowyj R.S., Gray D.G. (1980). Ordered phase formation in concentrated hydroxypropylcellulose solutions. Macromolecules.

[5] Charlet G., Gray D.G. (1987). Solid cholesteric films cast from aqueous (hydroxypropy1). Cellulose.

[6] Nishio Y., Kai T., Kimura N., Oshima K., Suzuki H. (1998). Controlling the selective light reflection of a cholesteric liquid crystal of (hydroxypropyl)cellulose by electrical stimulation. Macromolecules.

[7] Chiba R., Nishio Y., Miyashita Y. (2003). Electrooptical behavior of liquid-crystalline (hydroxypropyl)cellulose/inorganic salt aqueous solutions. Macromolecules.

[8] Nishio Y., Chiba R., Miyashita Y., Oshima K., Miyajima T., Kimura N., Suzuki H. (2002). Salt addition effects on mesophase structure and optical properties of aqueous hydroxypropyl cellulose solutions. Polym. J..

[9] Shimamoto S., Uraki Y., Sano Y. (2000). Optical properties and photopolymerization of liquid crystalline (acetyl) (ethyl) cellulose/acrylic acid system. Cellulose.

[10] Guo J.X., Gray D.G. (1994). Effect of degree of acetylation and solvent on the chiroptical properties of lyotropic (acetyl)(ethyl) cellulose solutions. J. Polym. Sci. Part B Polym. Phys..

[11] Gray D.G. (1994). Chiral nematic ordering of polysaccharides. Carbohydr. Polym..

[12] Ito M., Teramoto Y., Nishio Y. (2012). Electrooptical behavior of aqueous (hydroxypropyl)cellulose liquid crystals containing imidazolium salts. Biomacromolecules.

[13] Chiba R., Ito M., Nishio Y. (2010). Addition effects of imidazolium salts on mesophase structure and optical properties of concentrated hydroxypropylcellulose aqueous solutions. Polym. J..

[14] Muller M., Zentel R. (2000). Cholesteric phases and films from cellulose derivatives. Macromol. Chem. Phys..

[15] Wenzlik D., Zentel R. (2013). High optical quality films of liquid crystalline cellulose derivatives in acrylates. Macromol. Chem. Phys..

[16] Bhadani S.N., Gray D.G. (1984). Crosslinked cholesteric network from the acrylic-acid ester of (hydroxypropyl) cellulose. Mol. Cryst. Liq. Cryst..

[17] Muller M., Zentel R., Keller H. (1997). Solid opalescent films originating from urethanes of cellulose. Adv. Mater..

[18] Zhao C.T., Cai B.L. (1995). UV-Initiated solidification of liquid crystalline ethylcellulose/acrylic acid films and bands formed in this process. Macromol. Rapid Commun..

[19] Wenzlik D., Varanytsia A., Munoz A., Kosa T., Taheri B., Zentel R., Palffy-Muhoray P. (2014). Distributed feedback lasing in cellulose films. Opt. Mater. Express.

[20] Wenzlik D., Ohm C., Serra C., Zentel R. (2011). Preparation of cholesteric particles from cellulose derivatives in a microfluidic setup. Soft Matter.

[21] Shopsowitz K.E., Qi H., Hamad W.Y., MacLachlan M.J. (2010). Free-standing mesoporous silica with tunable chiral nematic structures. Nature.

[22] Mostofa K., Hamad W.Y., MacLachlan M.J. (2014). Tunable mesoporous bilayer photonic resins with chiral nematic structures and actuator properties. Adv. Mater..

[23] Klemm D., Friederike K., Moritz S., Lindstrom T., Ankerfors M., Gray D., Dorris A. (2011). Nanocelluloses: A new family of nature-based materials. Angew. Chem. Int. Ed..

[24] Fernandes S.N., Geng Y., Vignolini S., Glover B.J., Trindade A.C., Canejo J.P., Almeida P.L., Brogueira P., Godinho M.H. (2013). Structural color and iridescence in transparent sheared cellulosic films. Macromol. Chem. Phys..

[25] Habibi Y. (2014). Key advances in the chemical modification of nanocelluloses. Chem. Soc. Rev..

[26] Gaspar D., Fernandes S.N., de Oliveira A.G., Fernandes F.G., Grey P., Pontes R.V., Pereira L., Martins R., Godinho M.H., Fortunato E. (2014). Nanocrystalline cellulose applied simultaneously as the gate dielectric and the substrate in flexible field effect transistors. Nanotechnology.

[27] Geng Y., Brogueira P., Figueirinhas J.L., Godinho M.H., Almeida P.L. (2013). Light shutters from nanocrystalline cellulose rods in a nematic liquid crystal. Liq. Cryst..

[28] Godinho M.H., Fonseca J.G., Ribeiro A.C., Melo L.V., Brogueira P. (2002). Atomic force microscopy study of hydroxypropylcellulose films prepared from liquid crystalline aqueous solutions. Macromolecules.

[29] Sena C., Godinho M.H., Oliveira C.L.P., Neto A.M.F. (2011). Liquid crystalline cellulosic elastomers: Free standing anisotropic films under stretching. Cellulose.

[30] Godinho M.H., Filip D., Costa I., Carvalho A.-L., Figueirinhas J.L., Terentjev E.M. (2009). Liquid crystalline cellulose derivative elastomer films under uniaxial strain. Cellulose.

[31] Canejo J.P., Borges J.P., Godinho M.H., Brogueira P., Teixeira P.I.C., Terentjev E.M. (2008). Helical twisting of electrospun liquid crystalline cellulose micro- and nanofibres. Adv. Mater..

[32] Canejo J.P., Godinho M.H. (2013). Cellulose perversions. Materials.

[33] Godinho M.H., Canejo J.P., Feio G., Terentjev E.M. (2010). Self-winding of helices in plant tendrils and cellulose liquid crystal fibres. Soft Matter.

[34] Guo J.X., Gray D.G. (1989). Preparation and liquid-crystalline properties of (acetyl)(ethyl) cellulose. Macromolecules.

[35] Bertails F., Audoly B., Cani M.-P., Querleux B., Leroy F., Leveque J.-L. (2006). Super-helices for predicting the dynamics of natural hair. ACM Trans. Graph..

[36] Goriely A., Tabor M. (1998). Spontaneous helix hand reversal and tendril perversion in climbing plants. Phys. Rev. Lett..

[37] Hongladarom K., Secakusuma V., Burghardt W.R. (1994). Relation between molecular orientation and rheology in lyotropic hydroxypropylcellulose solutions. J. Rheol..

[38] Riti J.B., Navard P. (1998). Textures during recoil of anisotropic hydroxypropylcellulose solutions. J. Rheol..

[39] Kundu S., Feio G., Pinto L.F.V., Almeida P.L., Figueirinhas J.L., Godinho M.H. (2010). Deuterium NMR study of orientational order in cellulosic network microfibres. Macromolecules.

[40] Hongladarom K., Ugaz V.M., Cinader D.K., Burghardt W.R., Quintana J.P., Hsiao B.S., Dadmun M.D., Hamilton W.A., Butler P.D. (1996). Birefringence, X-ray scattering, and neutron scattering measurements of molecular orientation in sheared liquid crystal polymer solutions. Macromolecules.

[41] Keates P., Mitchell G.R., Peuvrel D.E., Navard P. (1993). Insitu X-ray-scattering study of anisotropic solutions of hydroxypropylcellulose subjected to shear-flow. Polymer.

[42] Geng Y., Almeida P.L., Feio G.M., Figueirinhas J.L., Godinho M.H. (2013). Water-Based Cellulose Liquid Crystal System Investigated by Rheo-NMR. Macromolecules.

[43] Godinho M.H., van der Klink J.J., Martins A.F. (2003). Shear-history dependent “equilibrium” states of liquid-crystalline hydroxypropylcellulose solutions detected by rheo-nuclear magnetic resonance. J. Phys.-Condensed Matter.

[44] Burghardt W.R., Fuller G.G. (1991). Role of director tumbling in the rheology of polymer liquid crystal solutions. Macromolecules.

[45] Marrucci G. (1991). Tumbling regime of liquid-crystalline polymers. Macromolecules.

[46] Onogi S., Asada T. (1980). Rheology and rheoptics of polymer liquid crystals. Rheology.

[47] Grabowski D.A., Schmidt C. (1994). Simultaneous measurement of shear viscosity and director orientation of a side-chain liquid-crystalline polymer by Rheo-NMR. Macromolecules.

[48] Samuels R.J. (1969). Solid-state characterization of the structure and deformation behavior of water-soluble hydroxypropylcellulose. J. Polym. Sci. Part A 2 Polym. Phys..

[49] Evmenenko G., Yu C.J., Kewalramani S., Dutta P. (2004). Structural characterization of thin hydroxypropylcellulose films. X-ray reflectivity studies. Langmuir.

[50] Patnaik S.S., Bunning T.J., Adams W.W., Wang J., Labes M.M. (1995). Atomic-force microscopy and high-resolution scanning electron-microscopy study of the banded surface-morphology of hydroxypropylcellulose thin-films. Macromolecules.

[51] Nishio Y., Takahashi T. (1984). Morphological study of hydroxypropyl cellulose films prepared from thermotropic melt under shear. J. Macromol. Sci. Phys..

[52] Mori N., Morimoto M., Nakamura K. (1999). Hydroxypropylcellulose films as alignment layers for liquid crystals. Macromolecules.

[53] Wang J., Labes M.M. (1992). Control of the anisotropic mechanical-properties of liquid-crystal polymer-films by variations in their banded texture. Macromolecules.

[54] Wang J., Bhattacharya S., Labes M.M. (1991). Solvent evaporation induced torsad texture of sheared liquid-crystalline polymers. Macromolecules.

[55] Geng Y., Almeida P.L., Fernandes S.N., Cheng C., Palffy-Muhoray P., Godinho M.H. (2013). A cellulose liquid crystal motor: A steam engine of the second kind. Sci. Rep..

[56] Bladon P., Warner M., Terentjev E.M. (1994). Orientational order in strained nematic networks. Macromolecules.

[57] Andresen E.M., Mitchell G.R. (1998). Orientational behaviour of thermotropic and lyotropic liquid crystal polymer systems under shear flow. Europhys. Lett..

[58] Favier V., Canova G.R., Cavaille J.Y., Chanzy H., Dufresne A., Gauthier C. (1995). Nanocomposite materials from latex and cellulose whiskers. Polym. Adv. Technol..

[59] Almeida P.L., Kundu S., Beja D., Fonseca J., Figueirinhas J.L., Godinho M.H. (2009). Deformation of isotropic and anisotropic liquid droplets dispersed in a cellulose liquid crystalline derivative. Cellulose.

[60] Borges J.P., Godinho M.H., Martins A.F., Trindade A.C., Belgacem M.N. (2001). Cellulose based composite films. Mech. Compos. Mater..

[61] Borges J.P., Godinho M.H., Belgacem M.N., Martins A.F. (2001). New bio-composites based on short fibre reinforced hydroxypropylcellulose films. Compos. Interfaces.

[62] Craighead H.V., Cheng J., Hackwood S. (1982). New display based on electrically induced index matching in an inhomogeneous medium. Appl. Phys. Lett..

[63] Godinho M.H., Figueirinhas J.L., Martins A.F. (1996). Novel PDLC type display based on cellulose derivatives. Liq. Cryst..

[64] Godinho M.H., Martins A.F., Figueirinhas J.L. (1998). Composite systems for display applications from cellulose elastomers and nematic liquid crystals. Opt. Mater..

[65] Almeida P.L., Godinho M.H., Cidade M.T., Figueirinhas J.L. (2001). Electro-optical properties of cellulose based PDLC type cells: Dependence on the type of diisocyanate cross-linking agent used. Mol. Cryst. Liq. Cryst..

[66] Almeida P.L., Kundu S., Borges J.P., Godinho M.H., Figueirinhas J.L. (2009). Electro-optical light scattering shutter using electrospun cellulose-based nano- and microfibers. Appl. Phys. Lett..

[67] Kato T. (2002). Self-assembly of phase-segregated liquid crystal structures. Science.

